# The association of cancer survival with four socioeconomic indicators: a longitudinal study of the older population of England and Wales 1981–2000

**DOI:** 10.1186/1471-2407-7-20

**Published:** 2007-01-25

**Authors:** Andrew Sloggett, Harriet Young, Emily Grundy

**Affiliations:** 1 London School of Hygiene and Tropical Medicine, London, UK

## Abstract

**Background:**

Many studies have found socioeconomic differentials in cancer survival. Previous studies have generally demonstrated poorer cancer survival with decreasing socioeconomic status but mostly used only ecological measures of status and analytical methods estimating simple survival. This study investigate socio-economic differentials in cancer survival using four indicators of socioeconomic status; three individual and one ecological. It uses a relative survival method which gives a measure of excess mortality due to cancer.

**Methods:**

This study uses prospective record linkage data from The Office for National Statistics Longitudinal Study for England and Wales. The participants are Longitudinal Study members, recorded at census in 1971 and 1981 and with a primary malignant cancer diagnosed at age 45 or above, between 1981 and 1997, with follow-up until end 2000. The outcome measure is relative survival/excess mortality, compared with age and sex adjusted survival of the general population. Relative survival and Poisson regression analyses are presented, giving models of relative excess mortality, adjusted for covariates.

**Results:**

Different socioeconomic indicators detect survival differentials of varying magnitude and definition. For all cancers combined, the four indicators show similar effects. For individual cancers there are differences between indicators. Where there is an association, all indicators show poorer survival with lower socioeconomic status.

**Conclusion:**

Cancer survival differs markedly by socio-economic status. The commonly used ecological measure, the Carstairs Index, is adequate at demonstrating socioeconomic differentials in survival for combined cancers and some individual cancers. A combination of car access and housing tenure is more sensitive than the ecological Carstairs measure at detecting socioeconomic effects on survival – confirming Carstairs effects where they occur but additionally identifying effects for other cancers. Social class is a relatively weak indicator of survival differentials.

## Background

The objective of this study is to investigate socio-economic differentials in cancer survival in England and Wales using four indicators of socioeconomic status; three individual and one ecological. Previous studies have generally demonstrated poorer cancer survival with decreasing socioeconomic status but often used only ecological measures of status, or less extensive analytical methods.

Most recent work on cancer survival in England and Wales has used patient data from cancer registries, which are considered of good quality and near-complete coverage. A major reference work on cancer survival, Cancer Survival Trends (CST) in England and Wales: 1971 – 1995, was published in 1999[1]. This work was detailed and comprehensive in its coverage of survival and a finding of considerable policy importance was a gradient in survival across levels of disadvantage for many cancers. An ecological indicator of disadvantage, the Carstairs index, was used because cancer registry data has poor or non-existent recording of individual socioeconomic or sociodemographic variables. An analogous study for Scotland showed similar results[2].

The Carstairs index is a composite score of levels of disadvantage in a defined geographic area, as measured by four components: male unemployment, overcrowded households, household car access, low social class of heads of households[3]. Although such measures have known limitations they have nevertheless proved extremely useful where individual level data are lacking[1,2,4-8].

The Office for National Statistics Longitudinal Study (ONS LS) provides an opportunity to investigate socio-economic differences in survival using variables measured at the individual level. The LS is a record linkage study providing census information, cancer registration and death registration data for an effectively random 1% sample of the population of England and Wales (the sample members). The original sample was drawn from people enumerated in the 1971 Census and has been maintained through the addition of 1% of immigrants and new births and so remains nationally representative. Information from later censuses was added as it became available. Tracing of events such as cancer registration and death is achieved via the National Health Services Central Register. Details of tracing and linkage rates, which are high, have been reported elsewhere[9].

The LS is limited by being a 1% sample, but has a major advantage over cancer registry data because of the availability of individual socioeconomic and sociodemographic information. Previous cancer survival/mortality studies have used the LS but have been less extensive than the present study and have not made use of relative survival models[10-12].

The use of relative survival for cancer studies is now well established[13]. The term implies survival of cancer patients relative to survival expected from age and sex matched rates for the general population. Where cause of death registration is accurate and complete cancer survival may be estimated directly by censoring non-cancer deaths in the analysis; results are often very similar to the relative survival approach. However, relative survival is considered more suitable because it calculates excess mortality following a diagnosis of cancer, even if direct cause of death is not cancer. This is more realistic for the many cases where cancer, or its treatment, is debilitating and causes death by the generation or worsening of other morbidities. Multivariate models of relative survival have been described and used in other studies, but not widely: perhaps due to their perceived complexity and the fact that some software cannot accommodate them[6,14-16].

## Methods

This study did not require full ethical approval as it comprises secondary analysis of established anonymised data. It was however approved by the Longitudinal Study Research Board of the Office for National Statistics.

The study sample selected comprised LS members with a first diagnosis of a primary malignant cancer at age 45 years or above, between 1981 and 1997. Additionally, those included in the analysis had to have been present at both the 1971 and 1981 Censuses. Registrations with a date of death on the same day as diagnosis (8.75% overall) were excluded as these are known to be mostly "death certificate only" diagnoses and are not usually included in survival analysis. In cases of multiple or synchronous registrations only the first occurring registration matching these criteria was selected.

Date of diagnosis and exit from the study were recorded. Date of exit was the earliest date of either death, emigration or end of study (31 December 2000). Dates were recorded to the day, thus giving survival durations in days.

Classification of cancer types followed the scheme presented in CST, updated where necessary for ICD-10. In this study "All cancers" refer to any registered primary malignancy, excluding non-melanoma skin cancer.

For modeling of relative survival individual records were split into survival segments each of a single year of follow-up. Up to five segments were used for each individual. The splitting of records allowed the correct updating of time-varying covariates such as age-group. Age-groups were: 45–49, 50–54, 55–59, 60–64, 65–69, 70–74, 75–79, 80+. The technique is described in detail elsewhere[16].

Calendar periods of diagnosis, or follow-up, were matched with general population mortality schedules for the calculation of relative survival. Population life tables were those published for 1980–82 and for 1990–92 for England and Wales, together with an inter-censal schedule centred on 1996 provided by the U.K. Government Actuaries Department[17]. The lifetables were therefore effectively centred on 1981, 1991 and 1996 and the relevant population risk of death, by age and sex, was matched with each survival segment depending on calendar period of survival: 1981–85, 1986–94,1995–2000 respectively.

Modeling of relative survival was conducted using Poisson regression of the counts of deaths, using a log offset of person-years at risk, for single years following diagnosis. A user-defined link function provided numbers of expected deaths, calculated from population life table risks, enabling rates relative to general population mortality to be modeled (*i.e*. rate ratios for relative excess mortality due to cancer)[14]. Because excess mortality is not constant by age it remains necessary to include adjustment for age in the regression models.

Socioeconomic differentials in survival were analysed using four indicators of socio-economic status available from the Census. These were, Registrar General's Social Class; housing tenure; household access to a car and the Carstairs indicator of relative area deprivation. Variables were those recorded at the census before diagnosis. Social class was unclassified for nearly a third of individuals, mostly women. In models where social class was the only socioeconomic variable it was used in an ordinal form and missing values were, of necessity, excluded. For models where social class is included with other socioeconomic variables as a control it was used in a categorical form such that missing values are retained as a separate category. For other socioeconomic indicators levels of missing values are much lower, at around 2%.

Socioeconomic variables are categorised in Table 1.

Additionally we included an indicator of geographic location at census. We first investigated using government region, this showed a simplified indicator of north/south zone was sufficient and this was used for simplicity. This splits England and Wales along a line extending from the Severn estuary to The Wash.

Analysis was performed using the statistical package Stata. Point estimates of crude and relative survival were made using the Stata ***strel ***routine derived for the CST study[1].

## Results

Rate ratios for relative excess mortality by different indicators of disadvantage are shown in Table 2. Model 1 gives rate ratios for the disadvantage indicator controlled for year of follow-up, age group, sex (where appropriate), period of diagnosis and north/south geographic zone. Model 2 additionally controls for other socioeconomic indicators shown in the table.

Table 2 demonstrates strong effects for all socioeconomic indicators, for all cancers combined. This finding persists in separate sex models.

For individual cancers there is a consistent suggestion, indicated by rate ratios greater than unity, of poorer survival with increasing disadvantage. This finding is not always confirmed statistically but for some sites (*i.e*. oesophagus) the consistency of raised rate ratios across indicators suggests that socioeconomic effects on mortality may be better confirmed in studies of higher power.

There are certainly differences between the extent of differentials identified. For individual cancers, Social Class is statistically weak. This may be because many individuals, mostly women, are unclassified by this scheme and therefore excluded from models where social class is the only socioeconomic indicator used; the analysis therefore has less power and may be subject to bias. The ecological Carstairs indicator is more powerful, confirming a socioeconomic gradient for cancer of the lung, breast and bladder.

The Car Access indicator shows a socioeconomic differential for five cancers: lung, colorectal, bladder, cervix/uterus and ovary, but not breast. Tenure shows a socioeconomic differential for six cancers: lung, colorectal, breast, bladder, leukaemia, ovary. Only for lung cancer is a differential confirmed by every indicator.

For combined cancers, the different socioeconomic indicators retain their significance in models including the other socioeconomic indicators (Model 2), with the sole exception of Social Class in the female model. In individual site models the Carstairs indicator retains its significance for lung and breast. For bladder, cervix/uterus and ovary the Carstairs indicator loses its significance whereas the Car Access indicator does not.

Survival differentials are particularly strong for bladder cancer. For cancer of ovary and cervix/uterus car access suggests a strong socioeconomic differential, even though this is not supported by other indicators. Table 3 summarises these findings.

To test the gender specificity of the indicators for separate cancers interaction terms were fitted. Few interactions were significant (likelihood ratio test) and no clear pattern of results was apparent. Interactions by period of diagnosis, investigating possible changes in sensitivity of indicators, were also inconclusive.

## Discussion

Different socioeconomic indicators detect survival differentials of varying magnitude and definition. For all cancers combined, the four indicators show similar effects. For individual cancers there are differences between indicators. But where there is an association, all indicators show poorer survival with lower socioeconomic status.

Many studies have demonstrated associations between socioeconomic indicators and mortality or survival following a cancer diagnosis, although others have inconclusive results[18].

Kogevinas *et al*, using the LS, found wide survival differentials between housing tenure groups[10]. A later LS study found rather weak effects of better survival among owner-occupiers, compared to tenants, for younger subjects suffering from breast, ovarian or prostate cancer.

Using data from the South Thames Cancer Registry, Schrijvers *et al *investigated relative survival from ten common cancers[6]. The Carstairs index was used as a proxy of socioeconomic disadvantage. They found poorer survival with increasing deprivation level of area for 7 out of the 10 cancers. Stomach, pancreas and ovarian cancer showed directionally similar gradients but these were weaker and insignificant in controlled models.

The largest and most detailed study of cancer survival in the UK to date is the CST study. Using the Carstairs index, it showed improved survival among those living in less-deprived areas. However, this was only confirmed statistically for 21 cancers[1]. Despite its size and comprehensive scope the CST study did not employ multivariate methods, indeed only some results were age-standardised. The Scottish study was more modest in scope but did test survival by age and sex controlled Cox models[2]. This also used the Carstairs score as a socio-economic indicator and gradients were found for many cancers.

McDavid *et al *conducted one of the few studies to date using controlled multivariate models of relative excess mortality to investigate cancer survival[15]. Survival from cancers of the breast, prostate, lung and colorectum in Kentucky were all strongly associated with type of health insurance held – itself related to socioeconomic status.

Greenwald *et al *suggested socioeconomic influence on survival may have separate mechanisms of action – survival differentials for highly lethal cancers may be the result of income differentials buying better care (in the US), whereas for cancers of better prognosis education differentials may affect disease progression[19]. Supporting this, an investigation into cancer survival in Turin, showed a strong positive relation between survival and higher levels of education for some less lethal cancers only[20].

Level of education is perhaps the missing socioeconomic indicator in this study. The British census has made various attempts to capture educational level in censuses and the questions asked in the 2001 census are very promising. However questions in the 1981 and 1991 censuses asked only about education after age 18. For the older cohorts in this study, especially women, this is even less useful than social class.

To our knowledge this is only the second application of multivariate models of relative excess mortality (the complement of relative survival) to British cancer data: previous studies using less sophisticated case fatality rates, Cox regression models or even simpler statistics. In addition our models are not only controlled for age and sex but also for period of diagnosis and geographic zone.

The fact that lifetables are not easily available by socioeconomic status and therefore national tables are used here for estimating relative survival could theoretically over-emphasise socioeconomic differentials. This effect, though recognized, is likely to be small and is largely obviated by socioeconomic controls in survival models[21]. Overall relative survival estimates obtained were very similar to those presented by the CST study, which did use specially constructed lifetables reflecting socioeconomic gradients.

The finding that combined cancers show strong socioeconomic gradients for survival is not quite as clear-cut as it appears because it is confounded by the fact that many of the cancers with higher incidence among the disadvantaged are those that are rapidly lethal (lung, oesophagus). Those that show slightly higher incidence in the more advantaged (breast, prostate) have longer survival periods. In other words the "case mix" of combined cancers varies with socioeconomic status and this affects survival.

All indicators suggest a socioeconomic gradient for lung cancer. This gradient is small but well defined and is perhaps an example of improved power of detection of such small effects for a common cancer. For other individual cancers there are differences between the indicators. Survival gradients by Social Class are generally not well defined, probably due to the known problems of unclassified individuals, especially older women.

The import of demonstrating survival differentials between groups with rate ratios in the order of 1.04 (lung) is questionable but is related to the speed of lethality of the cancer and may well reflect only lead time differences. For sites such as bladder and cervix/uterus, for which survival times can be lengthy, rate ratios approaching 2 between groups are substantial, important, and less likely to be merely lead time bias.

The Carstairs score picks out bladder and breast cancer as having a socioeconomic gradient for survival but only for bladder cancer do the car access and tenure indicators endorse this effect, showing a much stronger association than the ecological indicator. The effect concurs with recent findings for bladder cancer but may well have been missed had only the Carstairs score been used[22]. Car access and tenure detect an association for colorectal cancer. This is not particularly well defined but is not detected by the Carstairs measure at all. This is unexpected since the CST study found a noticeable gradient with Carstairs quintile, although their deprivation results were not age-standardised. However not all studies have found socioeconomic differentials in survival from this cancer and this association may well vary by indicator used[23].

For prostate and cervix/uterus a gradient by Carstairs' score of the local area is suggested in uncontrolled models (not shown) but not confirmed in controlled models. This suggests that at least some of the gradients identified in the CST study might not have persisted after age/sex adjustment. However the Scottish trends study does suggest a socioeconomic gradient (measured using Carstairs score) for age adjusted Cox models of prostate and cervix (but not uterus).

Survival gradients in combined leukaemias are difficult to demonstrate, probably because they are not a homogenous group of diseases. The Scottish study does not confirm a survival gradient by Carstairs quintile for the combined group but the larger CST study demonstrates a large gradient for chronic lymphoid leukaemia but very much smaller effects in other leukaemias.

The indicator with an interesting profile of results is car access: an indicator that is widely used in social science studies. It shows a socioeconomic differential for colorectal cancer and quite strong effects for cervix/uterus and ovary that are not shown by Carstairs quintile or particularly well by tenure. The effect for ovarian cancer is particularly interesting as it seems to be a strong effect. Although the Scottish and CST studies detect a significant gradient by Carstairs quintile it is not strong. These differences suggest that some indicators are particularly sensitive to differences in survival for particular cancers, possibly the more lethal women's cancers, and may be more useful in these cases.

Although there is undoubted correlation between the indicators used here it may well be that the utility of different indicators is their ability to select rather different social groupings. For instance no car access (in Britain) may be selecting a relatively small, particularly deprived, and possibly rural biased, group as well as the elderly.

It is tempting to explain socioeconomic differentials in survival by the simple explanation that patients of lower social class, or education, ignore symptoms and present later for diagnosis. Some studies have found less compliance with screening and more co-morbidity among certain groups, including the socially disadvantaged [24-29]. However in two studies using comprehensively controlled multivariate methods, adjustment for stage at diagnosis did nothing to change socioeconomic differentials[6,15]. Other studies have also failed to show definitive links between deprivation and cancer stage or biology or tumour size [23,26,27,30-32]. Therefore, although delayed presentation must play a part, it is by no means a complete explanation.

The fact that socioeconomic association with survival appears to be specific for certain cancers does not endorse any simplistic biological theory linking disease progression in general with poverty or disadvantage. In populations with largely equitable healthcare access this leaves differential treatment, health status and co-morbidity, coping and support strategies (embracing level of income), and understanding of, and ability to influence, disease progression (embracing educational level) as explanatory mechanisms for differential survival by socioeconomic status.

Ways of measuring socioeconomic status are manifold and their utility for older people is especially problematic[33]. Nevertheless it is important to consider different measures, especially when it is possible that the measures most widely used in cancer studies – the ecological measures – may not be the most sensitive.

## Conclusion

For all cancers combined four indicators of socioeconomic status show similar effects but for individual cancers there are different associations between socioeconomic indicators. Where there is an association all indicators show poorer survival with lower socioeconomic status.

Social class is a relatively weak indicator, not least because many individuals, especially older women, are unclassified by this scheme. A combination of car access and housing tenure seems more sensitive than the ecological Carstairs measure at detecting socioeconomic differentials in survival – confirming Carstairs effects where they occur (lung, bladder) but additionally identifying effects for cancers of the colorectum, cervix/uterus and ovary, and for leukaemias. For ovarian cancer particularly, reported in larger studies as having only a small socioeconomic gradient when measured by ecological measures, car access identifies a relatively strong socioeconomic effect on survival.

There is no clear evidence of sex-specificity of any socioeconomic indicator, nor of a changing association with period of diagnosis.

Researchers in the area of cancer survival should endeavour to collect individual or household level indicators of socioeconomic status when analysing survival and be aware that different indicators may be more specific for different cancers.

## Competing interests

The author(s) declare that they have no competing interests.

## Authors' contributions

AS designed the study, conducted the survival analysis and drafted the paper. HY extracted the data, advised on data formats and variable selection, undertook necessary data processing and preliminary analysis. EG advised on substantive aspects of the study and helped draft the discussion. All authors read and approved the final manuscript.

## Pre-publication history

The pre-publication history for this paper can be accessed here:



## Figures and Tables

**Table 1:** Categorisation of socioeconomic variables.

**Socioeconomic variable**	**Categorisation**	**Comparison**
Social Class	Registrar General's Social Class coded 1–6 for classes I-V respectively. All other values considered missing in models of social class as only s/e variable (column 1 of Table 2) and as a separate "other" category in other models.	Rate ratio per unit change in 6-point scale (professional to unskilled).
Carstairs quintiles	Carstairs index for wards of E&W. All residents of ward assigned quintile based on ward ranking.	Rate ratio per unit change in 5-point scale (less to more disadvantage).
Car Access	Access to car or van by household.	No car access *vs *any access.
Housing Tenure	Owner-occupation or rented accommodation	Rent *vs *own/occ.

**Table 2:** Rate ratio, with 95% Confidence Interval, for excess mortality by socioeconomic indicator.

**Cancer**		**Social Class Model**		**Carstairs Score Model**		**Car Access Model**		**Tenure Model**	
All cancers^†^ N = 26,273	1	**1.11**	**1.09–1.14**	**1.12**	**1.10–1.14**	**1.41**	**1.34–1.49**	**1.45**	**1.38–1.52**
	2	**1.05**	**1.02–1.08**	**1.06**	**1.04–1.08**	**1.19**	**1.13–1.26**	**1.26**	**1.20–1.33**
All cancers, Male N = 13,523	1	**1.11**	**1.08–1.14**	**1.11**	**1.09–1.14**	**1.45**	**1.35–1.55**	**1.44**	**1.34–1.53**
	2	**1.05**	**1.02–1.08**	**1.05**	**1.03–1.08**	**1.22**	**1.13–1.32**	**1.24**	**1.16–1.34**
All cancers, Female N = 12,750	1	**1.10**	**1.06–1.15**	**1.12**	**1.09–1.15**	**1.34**	**1.24–1.46**	**1.42**	**1.33–1.53**
	2	1.04	0.99–1.09	**1.07**	**1.04–1.10**	**1.14**	**1.05–1.24**	**1.27**	**1.17–1.37**
Lung N = 4,271	1	**1.04**	**1.01–1.08**	**1.06**	**1.03–1.09**	**1.13**	**1.05–1.22**	**1.11**	**1.03–1.19**
	2	1.02	0.99–1.06	**1.04**	**1.01–1.08**	1.07	0.98–1.17	1.04	0.95–1.12
Oesophagus N = 608	1	**1.12**	**1.01–1.24**	1.07	0.99–1.16	1.21	0.96–1.51	1.14	0.91–1.42
	2	1.11	0.99–1.23	1.06	0.98–1.15	1.10	0.86–1.41	1.06	0.84–1.33
Stomach N = 1,283	1	0.99	0.92–1.06	1.03	0.97–1.09	1.13	0.96–1.34	1.04	0.89–1.22
	2	0.99	0.90–1.05	1.02	0.96–1.09	1.11	0.93–1.33	0.99	0.83–1.17
Colorectal N = 3,298	1	1.01	0.94–1.08	1.01	0.96–1.07	**1.25**	**1.08–1.46**	**1.15**	**1.00–1.32**
	2	0.98	0.91–1.06	0.99	0.94–1.04	**1.24**	**1.05–1.46**	1.08	0.93–1.26
Female Breast N = 3,102	1	0.94	0.84–1.05	**1.14**	**1.06–1.22**	1.13	0.91–1.40	**1.28**	**1.06–1.55**
	2	0.90	0.80–1.01	**1.13**	**1.05–1.22**	0.97	0.77–1.21	1.17	0.95–1.45
Bladder N = 1,407	1	1.10	0.93–1.30	**1.22**	**1.06–1.40**	**2.30**	**1.52–3.48**	**2.01**	**1.41–2.87**
	2	0.95	0.79–1.15	1.14	0.98–1.32	**1.87**	**1.22–2.88**	**1.67**	**1.16–2.42**
Prostate N = 1,714	1	1.02	0.92–1.14	1.09	0.99–1.20	1.25	0.94–1.67	1.14	0.86–1.51
	2	0.99	0.88–1.11	1.07	0.97–1.18	1.14	0.83–1.57	1.02	0.76–1.38
Pancreas N = 641	1	1.09	0.99–1.21	1.02	0.94–1.09	1.06	0.85–1.33	1.13	0.91–1.41
	2	1.08	0.98–1.20	1.01	0.93–1.09	0.97	0.75–1.25	1.11	0.87–1.42
Leukaemia N = 446	1	1.07	0.87–1.32	1.09	0.95–1.24	1.25	0.80–1.96	**1.58**	**1.08–2.32**
	2	1.05	0.84–1.31	1.05	0.91–1.21	1.08	0.68–1.71	1.42	0.93–2.17
Cervix/Uterus N = 816	1	0.96	0.76–1.20	1.12	0.97–1.30	**1.91**	**1.29–2.81**	1.23	0.85–1.78
	2	0.95	0.75–1.21	1.08	0.92–1.26	**1.98**	**1.24–3.14**	0.85	0.55–1.32
Ovary N = 574	1	1.11	0.98–1.26	1.04	0.96–1.14	**1.59**	**1.23–2.05**	**1.34**	**1.05–1.71**
	2	1.03	0.90–1.18	0.99	0.91–1.09	**1.50**	**1.14–1.97**	1.21	0.93–1.57

Social Class model: Registrar-General's Social Class – six point scale.

Carstairs model: quintiles of Carstairs index – five point scale.

Car Access model: binary indicator of no household access to car/van.

Tenure model: binary indicator of renting accommodation.

Model 1: Socioeconomic indicator controlled for year of follow-up, age group, sex (where appropriate), period of diagnosis, marital status, north/south geographic zone.

Model 2: Model 1, additionally controlled for all other socioeconomic indicators shown.

Results in bold type significant at 5% level or below.

† any malignancy, not restricted to groups listed below

Source: ONS Longitudinal Study

**Table 3:** Summary table showing strength of association of excess mortality with socioeconomic indicators.

Socioeconomic effects confirmed by more than one indicator	Socioeconomic effect apparent but displayed by one indicator only (indicator showing effect)		Socioeconomic effect suggested by simple rate ratio but not confirmed by controlled models
Bladder	Cervix/Uterus	(Car Access)	Stomach
Female Breast	All Leukaemias	(Tenure)	Pancreas
Lung	Oesophagus	(Social Class)	Prostate
Ovary			
Colorectal			

Source: ONS Longitudinal Study
